# The Web-Based Randomized Controlled Intervention as the Enhancer of Cancer Prevention

**DOI:** 10.3390/medicina55080434

**Published:** 2019-08-03

**Authors:** Maksymilian Gajda, Małgorzata Kowalska

**Affiliations:** Department of Epidemiology, School of Medicine in Katowice, Medical University of Silesia, Medyków 18 Str., 40-752 Katowice, Poland

**Keywords:** prevention, cancer, web-based intervention, interventional studies, diagnostic procedures uptake

## Abstract

*Background and Objectives*: Cancer is an unresolved public health issue in society. With the advent of the internet and the development in the technological sector, access to basic health-related information has become more frequent among patients and healthy individuals. The aim of this study is to evaluate the impact of educational intervention on the participant’s willingness to undergo selected medical procedures in case of suspected cancer. *Materials and Methods*: From 14 May 2015 to 13 November 2016, a total of 1118 who visited the Polish scientific website were enrolled in the ‘Polish Online Randomized Intervention aimed at Neoplasm Avoidance’ (PORINA) and randomized into two groups (educational and control). The original Cancer Knowledge Index (CKI) was used for the evaluation of participants’ cancer-related knowledge. They were asked to declare whether they would consent to undergo selected medical procedures necessary for cancer diagnosis or treatment. *Results*: Most participants declared their readiness to undergo abdominal ultrasonography, computed tomography, and a nevi excision. The most noticeable changes were found for potential consent to undergo breast fine needle aspiration, mammography and gastroscopy. *Conclusions*: The level of oncological knowledge has an impact on individual decision to consent to particular medical procedures. Conducted educational intervention has significantly increased the readiness to undergo selected medical procedures.

## 1. Introduction

According to the GLOBOCAN 2018 data, overall cancer incidence and mortality are increasing [1]. The relatively high mortality in Poland accompanied by lower incidence of disease compared to the situation observed in many European Union countries is alarming [1,2]. These circumstances strongly support the need to recognize cancer as a priority. It has been shown that about half of the 11 malignancies that are considered serious health problems can be prevented [3,4]. Early diagnosis of cancer and the implementation of appropriate treatment are determined, inter alia, by the patients’ appropriate level of health-oriented knowledge. Unfortunately, factors such as lack of knowledge, sense of embarrassment, cultural factors, beliefs inconsistent with the scientific facts, as well as common myths hinder prevention campaigns and may significantly delay patient effort in seeking medical advice, diagnosis and treatment [5,6,7,8,9]. There is the need to implement interventional programs aimed at improving knowledge and participation rates in screening for, cancers such as colorectal cancer or melanoma [10,11,12,13,14]. The reason for the need to implement these interventional programs is due to the fact that cancer, remains an important and still unresolved public health issue on a global scale (as evidenced by the previously mentioned increase in negative epidemiological indicators [1,2]). This prompts the search for more effective preventive methods. At the same time, the dynamic development of the new technology sector, including the internet, has provided more frequent source of basic knowledge about health and illness among patients and healthy individuals [15,16]. The capabilities of the Internet are increasingly being used by public health researchers [17]. Improving access to reliable information on cancers, especially their prevention, may be helpful, among others, in promoting healthy behaviors, positively influencing participation in necessary diagnostic tests. The aim of this study is to evaluate the impact of educational intervention on the tendency of the participants to undergo selected medical procedures in case of suspected cancer.

## 2. Materials and Methods

### 2.1. General Description of the Study

A more detailed description of PORINA study’s methodology is available in our previous paper on the PORINA study [18]. Moreover, the protocol was extensively described in a doctoral thesis and can be provided on request. Here we present the most important issues necessary to interpret the results related to the aim of this paper.

**Figure 1:**
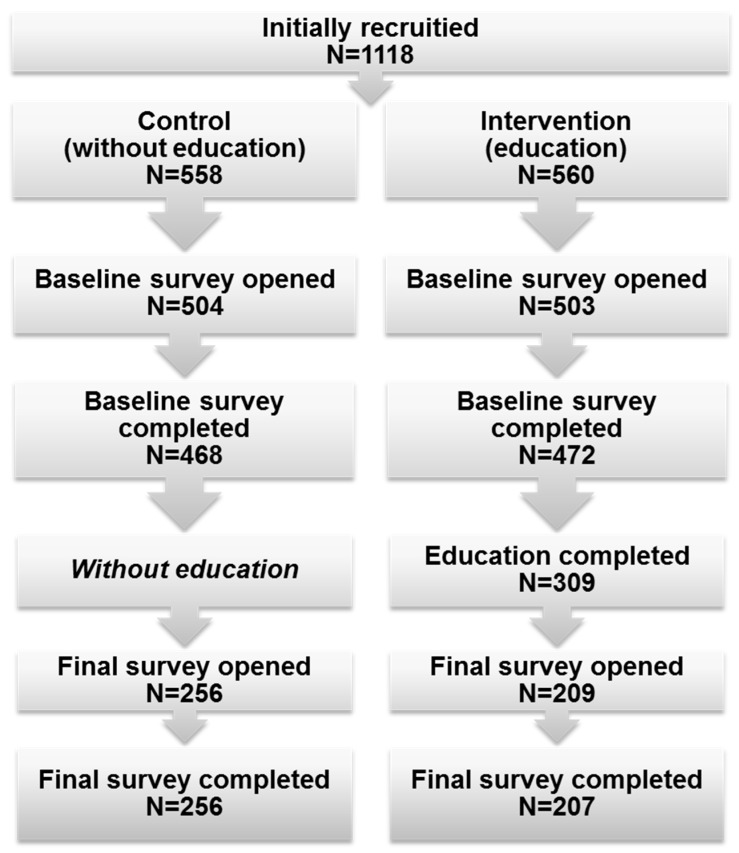
Summary of the study design and flow.

The study was conducted between 14 May 2015 and 13 November 2016. Individuals who visited one of the polish open-access scientific websites (‘Naukowy.pl’) were asked to participate in this prospective interventional study, the Polish On-line Randomized Intervention aimed at Neoplasm Avoidance (PORINA). We used a friendly advertisement displayed to the all visitors during the assumed study period. They were given all information about the study purposes and the way of conducting the research. Consent to participate in the study was in the electronic form. Voluntary consent and completion of all phases of the study were included among the most important inclusion criteria. Subjects not meeting all of inclusion criteria were not included in the final analysis. A dedicated internet platform was created to allow the study to be conducted (including retrieving informed consent in an electronic form). It allowed participants to be randomized into either control or interventional group. Those assigned to the interventional arm of the study were given the access to educational material, specifically prepared by a physician and were asked to fill the questionnaire before and after they got acquainted with the materials. After viewing the materials, the participants of the study were required to participate in a simple quiz. It enabled us to verify whether they had become acquainted with the educational module. Giving incorrect answers in the quiz made it impossible to complete the education phase and they were asked to get acquainted with the materials again. The control group were not given access to the educational module; they were only asked to fill the questionnaire twice allowing us to successfully validate the questionnaire, as presented in our former paper [18]. Figure 1 presents a simplified flowchart of the study design.

### 2.2. Methods

The author’s questionnaire survey prepared for the PORINA study was divided into two parts (i.e., demographical and cancer knowledge), containing only closed-ended questions written in Polish. The educational materials were prepared by the author—a physician who specializes in clinical oncology. The materials were accessible via the study online platform as an 11 min multimedia presentation narrated by the physician or a basic version consisted of text and images. Risk factors and prevention, the role of the alarm symptoms and the recommendations of the European Code Against Cancer were thoroughly discussed in the materials [19]. The original Cancer Knowledge Index (CKI) was used for the evaluation of participants’ cancer-related knowledge. This index was counted based on the responses to a set of questions, with each correct answer assigned 1 point. The adopted way of assigning points led to obtaining the maximum CKI value of 20 for the best and 0 for the worst knowledge. We distinguished three ranges of CKI values. “Low” included scores lower than the value of the first tertile. “Average” score denoted values between the first and second tertile (including their values). “High” scores comprised values above second tertile.

Participants were also asked to declare whether they would consent to undergo a presented list of medical procedures necessary for cancer diagnosis or treatment. Answers given to these questions were our main measures.

### 2.3. Data Analysis

Given the small size of some subgroups, the values (answers to the questions about preliminary consent to undergo selected medical procedures in cancer diagnosis or treatment) of ‘No’ and ‘I do not know’ were converted to one category of ‘No consent’, while the value ‘Yes’ was assigned to the ‘Consent’ category. This was done for the purposes of statistical analysis. For the same reasons, levels of education were combined in this manner: ‘Primary’ and ‘Secondary’ values were combined to form a single ‘Lower’ category, while both ‘High school’ and ‘High school medical’ were assigned to ‘Higher’ category. When interpreting the results it should be noted that “high school” corresponds to the master degree and above. A similar procedure was adopted for variable representing the place of residence. The two values, ‘Village’ and ‘City described as having ≤100,000 inhabitants’ were combined into one category of ‘≤100,000 inhabitants’, resulting in two categories: ‘≤100,000 inhabitants’ and ‘>100,000 inhabitants’ (in selected analysis).

The R software was used for statistical analysis [20]. The statistical significance threshold was assumed as *p*-values less than 0.05. The distribution of quantitative variables was assessed using the Shapiro–Wilk and Anderson–Darling tests. Differences between independent groups were assessed by U Mann-Whitney or ANOVA Kruskal–Wallis tests, while for categorical variables χ^2^ test was used. To evaluate the influence of educational intervention (expressed as changes in consents numbers) the McNemar test was used.

### 2.4. Ethical Approval

The protocol for this study was approved by the Bioethical Committee of the Medical University of Silesia in Katowice, Poland (KNW/0022/KB1/146/14; 24 December 2014).

## 3. Results

A total of 1118 participants were enrolled in the PORINA study, while 463 subjects who responded to all questions were included for the final analysis. Members of both study groups were similar in terms of demographics as well as family and individual history of cancer (Table 1).

We observed that higher level of education, age of subjects, medical occupation, and history of cancer can significantly affect subjects’ decision to undergo necessary medical procedures. In the case of educational level, the largest differences expressed in percentage changes were found for digital rectal examination ((DRE) almost 60%), colonoscopy (over 40%), and panendoscopy (near 30%). Patients with a cancer diagnosis were more likely to consent for surgery with possible artificial anus than healthy participants. Place of residence as well as gender were not significant determinants of potential consent, except for the abdominal ultrasonography (USG). Detailed results are presented in supplementary Table S1.

Statistical analysis with the McNemar test revealed that educational intervention significantly (*p* = 0.03) increased (by approximately 6%) in participants who affirmed consents for breast fine needle aspiration (BFNA) and mammography (MMG) (*p* = 0.004). The respondents stated that they are more likely to agree for endoscopic examination of the upper gastrointestinal tract (increase by 4.8%; *p* = 0.04). Changes for the rest of the procedures were not statistically significant. Detailed results are presented in Table 2. Detailed analysis showed significant increase in consents for BFNA and MMG procedures among participants who are male; non-medical occupation, without any family, or personal history of cancer (supplementary Table S2). Moreover, the increase of consents for BFNA, in contrary to MMG, was more frequent among lower educated participants and those living in less populated areas. Subjects, who declared their cancer-related knowledge as insufficient and expressed their willingness to improve it, more often consented for MMG and panendoscopy. Some statistically significant improvement should be stressed when it comes to DRE, but only for the participants living in less populated areas and those who are between 24 and 41 years old. No significant advantages were found in relation to agreement for abdominal USG, colonoscopy, CT, and surgical procedures.

## 4. Discussion

Participation in screening and other diagnostic procedures is one of the forms of preventive action for cancer [7,9]. Unfortunately, downplaying symptoms, and consequently late doctor visits, results in worse prognosis due to the higher stage of disease [21,22]. Published data provides evidence for the link between knowledge of the potential symptoms of cancer, postponement of a doctor’s visit, and survival time [5,23]. Other reasons for delayed counseling include fear of diagnosis, embarrassment, lack of time, age of patients, lack of courage to talk about symptoms during a medical visit, or even the sense of wasting a doctor’s time [7,22]. Meanwhile, current data from published online and traditional studies indicate that it is possible to increase the number of people involved in screening for breast, cervical and colorectal cancers [24,25,26]. Unfortunately, as the results of one study shows, knowledge improvement may not be the only factor responsible for changing a readiness to participate in screening for colorectal cancer [27]. Our study does not bring any additional explanations in this area, as it was not its aim. It has also been shown that online questionnaires with relevant feedback to participants after completion may provide important assistance in deciding to participate in the cancer screening programs [28]. The results of the PORINA study are consistent with published data. Participants subjected to educational intervention more frequently declared their willingness to undergo MMG and breast fine needle biopsy than the control. Bowen et al. (*n* = 334) reported a relative increase in MMG frequency by 15.8% and breast self-examination by as much as 35.5% [24]. Unfortunately, PORINA has not shown any significant change in readiness to undergo colonoscopy nor DRE. Participation rates in colorectal screening are still too low in many countries [8]. However, data from the 3rd cycle of Health Information National Trends Survey (HINTS 2008) project show a high level of awareness among respondents regarding the effectiveness of individual colorectal cancer diagnostic procedures: 86.7% of them considered colonoscopy while only 5.4% indicated fecal occult blood test as best screening method [29]. Education conducted during the PORINA study has increased the percentage of consent to possible upper gastrointestinal endoscopy uptake. The lowest number of participants declared the consent for surgical procedure with possible stoma forming. However, there was a slight increase in readiness to undergo this procedure, even though it was not statistically significant. As shown in supplementary Table S2, increases in consents gained with educational intervention mostly affected participants who declared an initial consent less often. The youngest participants had highest improve in the level of consents for MMG. In fact, this group is not a target population for MMG screening, due to the potentially of not being familiar with this medical procedure. However, improving preventive awareness at younger age could be beneficial during an appropriate stage of their life.

The results obtained indicated that young people are less likely to undertake majority of cancer-related diagnostic and medical procedures described above. Similarly, subjects with higher level of education and those with positive family history of cancer are more likely to participate in majority of screening tests. Gender and place of residence were not so important to declared decision, except for USG procedure.

### 4.1. Limitations of the Study

The authors are aware of other limitations of objective assessment of participants’ responses to the possible consent to undergo selected medical procedures, in addition to the inability to generalize the results (e.g., selection bias, especially volunteer bias). First, not every participant could have a sufficient knowledge about how a particular procedure is conducted. Lack of knowledge about the indications for treatment as well as possible complications were demonstrated during HINTS 4 (Health Information National Trends Survey 2014), in which only half of the respondents correctly indicated that the risks associated with screening (colonoscopy, MMG, and cytological screening) are far below the expected benefits [29]. Furthermore, the educational materials presented in the PORINA study did not directly address the individual medical procedures (including the methods, purpose, and possible complications). The importance of early diagnosis was rather emphasized. The results suggest that higher knowledge levels were associated with increased readiness to participate in most of the medical procedures that were included in the questionnaire. The largest diversity of responses was observed with respect to DRE and colonoscopy. In this case, a rare declaration of potential consent coincided with low awareness of the colorectal cancer screening availability.

### 4.2. The Practical Importance of the Scientific Report

Despite the limitations discussed previously, the results obtained are interesting and promising for public health, especially for institutions involved in the design and evaluation of health programs aimed at prevention. They provide evidence that web-based educational intervention may yield tangible benefits in the form of increased cancer-related knowledge of the internet users in Poland, especially in the field of prevention capabilities. It is the hope of public health professional that increased awareness will encourage early diagnosis and thus provide a more effective treatment and, consequently, a reduction in the specific mortality from cancer in Poland and other countries. More research is needed so that the discussed limitations could be addressed. As a natural consequence of the results obtained so far, the “real-life” impact of web-based intervention on actual participation in screening procedures should be evaluated in randomized controlled trial (not just the declaration of potential consent). Moreover, other possible conditions for avoiding screening (e.g., psychosocial influences) should be investigated.

## 5. Conclusions

The level of oncological knowledge has an impact on the decision to consent to undergo diagnostic or therapeutic medical procedures in the case of suspected cancer. The web-based educational intervention has significantly increased the frequency of preliminary consents to undergo selected medical procedures which are breast fine needle aspiration, mammography and gastroscopy. Although the potential of supporting cancer prevention with the use of online educational interventions seems promising, this topic requires further research.

## Figures and Tables

**Table 1:** Descriptive characteristics of subjects in the interventional and control groups.

				Group		
Quantitative Variables (Median and IQR in the Bracket)				Control	Intervention	*p*
				*n* = 256	*n* = 207	
Age (years)				31 (22–47)	35 (21–47)	0.9 ^a^
Baseline CKI				14 (12–16)	14 (11–16)	0.2 ^a^
Final CKI				14 (12–16)	17 (15–18)	<0.001 ^a^
Categorical variables (%)						
Gender		Male		39.1	38.2	0.8 ^b^
		Female		60.9	61.8	
Place of residence with the number of inhabitants		*Villages*		23.4	19.8	0.6 ^b^
		*Cities*	≤100,000	25.0	25.6	
			>100,000	51.6	54.6	
Level of education		Primary		4.7	8.2	0.2 ^b^
		Secondary		36.3	35.7	
		High school		51.2	51.7	
		Higher medical		7.8	4.3	
Occupation		Other		84.0	83.6	0.9 ^b^
		Medical		16.0	16.4	
Positive family history of cancer	overall	No		23.8	28.5	0.3 ^b^
		Yes		76.2	71.5	
	parents	No		70.3	73.9	0.4 ^b^
		Yes		29.7	26.1	
	grandparents	No		55.1	52.7	0.6 ^b^
		Yes		44.9	47.3	
	siblings	No		94.1	96.1	0.4 ^b^
		Yes		5.9	3.9	
Diagnosis of cancer		No		87.9	90.8	0.4 ^b^
		Yes		12.1	9.2	
Treated oncologically		No		89.5	89.9	1 ^b^
		Yes		10.5	10.1	
Self-declaration of sufficient cancer-related level of knowledge		No		78.5	77.8	0.9 ^b^
		Yes		21.5	22.2	
Self-declaration of willingness to improve the level of cancer-related knowledge		No		7.8	7.2	0.9 ^b^
		Yes		92.2	92.8	

IQR—interquartile range; *n*—number of subjects; *p*—statistical significance; ^a^—U Mann–Whitney test; ^b^—χ^2^ test.

**Table 2:** Differences in the frequency of declared consent to undergo selected medical procedures depending on (**A**) value of the Cancer Knowledge Index (CKI) and (**B**) influence of educational intervention.

A. Potential Agreement (%) Depending on Baseline CKI Value								
Medical Procedure	Overall	Low		Medium	High			*p* *
		(*n* = 107)		(*n* = 234)	(*n* = 122)			
Breast Fine Needle Aspiration	81.9	64.5		84.2	92.6			<0.001
Abdominal ultrasonography	98.1	95.3		98.3	100			<0.05
Mammography	88.1	82.2		86.8	95.9			<0.001
Digital Rectal Examination	79.0	61.7		81.2	90.2			<0.001
Colonoscopy	85.7	69.2		88.9	94.3			<0.001
Bronchoscopy	82.3	67.3		84.6	91.0			<0.001
Gastroscopy	85.3	72.0		87.6	92.6			<0.001
Removal of a nevi from the skin	89.8	74.8		92.7	97.5			<0.001
Computed Tomography	98.5	97.2		98.3	100			NS
Surgical procedure with possible artificial anus	47.3	29.0		51.3	55.7			<0.001
**B. Potential Agreement (%) Depending on Study Group**								
**Medical Procedure**	**Intervention**			*p* **	**Control**			*p* **
	**Initial**	**Final**	**Change**		**Initial**	**Final**	**Change**	
Breast Fine Needle Aspiration	81.2	87.4	6.2	<0.05	82.4	82.8	0.4	NS
Abdominal ultrasonography	98.1	99.0	0.9	NS	98.0	99.2	1.2	NS
Mammography	86.5	92.8	6.3	<0.01	89.5	91.4	1.9	NS
Digital Rectal Examination	76.3	80.7	4.4	NS	81.2	82.0	0.8	NS
Colonoscopy	83.6	86.5	2.9	NS	87.5	87.5	0.0	NS
Bronchoscopy	80.2	84.5	4.3	NS	84.0	84.8	0.8	NS
Gastroscopy	83.6	88.4	4.8	<0.05	86.7	88.3	1.6	NS
Removal of a nevi from the skin	87.4	88.9	1.5	NS	91.8	93.8	2.0	NS
Computed Tomography	98.1	97.6	−0.5	NS	98.8	98.0	−0.8	NS
Surgical procedure with possible artificial anus	44.9	49.3	4.4	NS	49.2	48.4	−0.8	NS

*n*—number of subjects; NS—not significant; *p* *—statistical significance in χ^2^ test; *p* **—statistical significance in McNemar test.

## Supplementary Materials

The following are available online at https://www.mdpi.com/1010-660X/55/8/434/s1. Table S1: Percentage of initial consents for selected medical procedures in relation to particular independent variables and statistical significance of differences in χ^2^ test, Table S2: Differences in percentages of consents for selected medical procedures in which was observed changes after educational intervention in relation to particular independent variables.
