# Supplementary material for: The Web-Based Randomized Controlled Intervention as the Enhancer of Cancer Prevention
Source: Medicina (Kaunas). 2019 Aug 3;55(8):434. doi: 10.3390/medicina55080434 (PMC6723358; doi:10.3390/medicina55080434)
Supplement: Supplementary file 1 [file medicina-55-00434-s001.pdf]

Table 1. Percentage of initial consents for selected medical procedures in relation to particular independent variables and statistical significance of differences in χ^2 test.

**Independent variables**		**BFNA** %	**p**	**USG** %	**p**	**MMG** %	**p**	DRE %	p	Col. %	p	Bron. %	p	Pan. %	p	Exc. %	p	CT %	p	Surgery %	p
Age group (years)	<24	77.0		96.7		89.5		64.5		72.4		75.7		76.3		93.4		96.7		34.9	
	24-41	81.9	NS	97.4	NS	87.7	NS	83.2	a	89.7	a	83.9	c	86.5	a	90.3	NS	99.4	NS	47.1	a
	>41	86.5		100.0		87.2		89.1		94.9		87.2		92.9		85.9		99.4		59.6	
Gender	Male	79.3	NS	95.5	b	88.8	NS	80.4	NS	84.4	NS	82.7	NS	86.6	NS	88.3	NS	97.2	NS	48.0	NS
	Female	83.5		99.6		87.7		78.2		86.6		82.0		84.5		90.8		99.3		46.8	
Number of inhabitants in place of residence	≤100,000	78.0	NS	96.3	c	87.6	NS	75.7	NS	82.1	NS	78.4	NS	82.1	NS	88.5	NS	97.7	NS	44.5	NS
	>100,000	85.3		99.6		88.6		82.0		89.0		85.7		88.2		91.0		99.2		49.8	
Level of education	Primary	69.0	b	89.7	b	86.2	NS	37.9	a	58.6	a	65.5	b	65.5	a	89.7	NS	89.7	a	31.0	c
	Secondary	76.0		98.2		89.2		74.3		81.4		77.2		80.8		88.0		98.2		40.7	
	Highschool	85.7		98.7		86.6		85.3		90.3		87.0		89.9		89.9		99.6		52.9	
	Higher medical	96.6		100.0		96.6		96.6		100.0		89.7		93.1		100.0		100.0		55.2	
Occupation	Other	80.2	c	97.7	NS	86.6	c	77.1	c	84.3	NS	81.2	NS	84.0	NS	88.4	c	98.2	NS	47.4	NS
	Medical	90.7		100.0		96.0		89.3		93.3		88.0		92.0		97.3		100.0		46.7	
Positive family history of cancer	No	70.0	a	96.7	NS	70.0	a	83.3	NS	87.5	NS	83.3	NS	86.7	NS	81.7	b	98.3	NS	46.7	NS
	Yes	86.0		98.5		94.5		77.6		85.1		81.9		84.8		92.7		98.5		47.5	
Participant with diagnosis of cancer	No	81.4	NS	98.1	NS	88.1	NS	78.2	NS	84.3	b	81.4	NS	84.5	NS	90.1	NS	98.5	NS	44.8	b
	Yes	86.0		98.0		88.0		86.0		98.0		90.0		92.0		88.0		98.0		68.0	
Participant treated oncologically	No	81.7	NS	98.1	NS	88.4	NS	77.6	c	84.6	c	81.2	NS	84.3	NS	90.1	NS	98.3	NS	44.6	a
	Yes	83.3		97.9		85.4		91.7		95.8		91.7		93.8		87.5		100.0		70.8	
Self-declaration of sufficient cancer-related level of knowledge	No	80.4	NS	97.5	NS	87.8	NS	77.3	NS	83.4	b	81.2	NS	84.3	NS	88.7	NS	98.1	NS	46.7	NS
	Yes	87.1		100.0		89.1		85.1		94.1		86.1		89.1		94.1		100.0		49.5	
Self-declaration of willingness to improve the level of cancer-related knowledge	No	77.1	NS	97.1	NS	88.6	NS	77.1	NS	85.7	NS	88.6	NS	88.6	NS	88.6	NS	97.1	NS	51.4	NS
	Yes	82.2		98.1		88.1		79.2		85.7		81.8		85.0		90.0		98.6		47.0	

BFNA–Breast Fine Needle Aspiration; USG–abdominal ultrasonography; Col.–colonoscopy; Bron.– bronchoscopy; Pan.– panendoscopy; Exc. –excision of a naevi; CT–computed tomography; Surg.- Surgical procedure with possible artificial anus; p – statistical significance in χ^2 test: a <0.001; b <0.01; c <0.05; NS–not significant.

Table 2. Differences in percentages of consents for selected medical procedures in which was observed changes after educational intervention in relation to particular independent variables.

**Independent variables**		Medical procedure													
		BFNA		USG		MMG		DRE		Col.		Bron.		Pan.	
		D	p	D	p	D	p	D	p	D	p	D	p	D	p
Age group (years)	<24	10.1	NS	0.0	NS	10.2	<0.05	7.3	NS	4.4	NS	7.2	NS	5.8	NS
	24-41	6.3	NS	0.0	NA	1.6	NS	9.5	<0.05	1.6	NS	1.5	NS	1.6	NS
	>41	2.6	NS	3.2	NS	6.7	NS	-2.7	NS	2.6	NS	4.0	NS	6.6	NS
Gender	Male	10.2	<0.05	1.3	NS	7.6	<0.05	3.8	NS	3.8	NS	6.4	NS	6.3	NS
	Female	3.9	NS	0.8	NA	5.5	NS	4.6	NS	2.3	NS	3.1	NS	3.9	NS
Number of inhabitants in place of residence	≤100,000	11.7	<0.05	3.2	NA	6.4	NS	8.6	<0.05	4.2	NS	6.4	NS	6.4	NS
	>100,000	1.8	NS	-0.9	NS	6.2	<0.05	0.9	NS	1.8	NS	2.6	NS	3.5	NS
Level of education	Lower	14.3	<0.01	1.1	NS	6.6	NS	5.5	NS	0.0	NS	5.5	NS	5.5	NS
	Higher	0.0	NS	0.8	NS	6.0	NS	3.4	NS	5.2	NS	3.5	NS	4.3	NS
Occupation	Other	7.5	<0.05	1.1	NS	5.8	<0.05	4.6	NS	4.1	NS	6.4	<0.05	6.4	<0.05
	Medical	0.0	NS	0.0	NA	8.8	NA	3.0	NS	-3.0	NS	-5.9	NS	-2.9	NS
Positive family history of cancer	No	15.3	<0.05	1.7	NS	16.9	<0.01	0.0	NS	3.4	NS	6.7	NS	8.4	NS
	Yes	2.7	NS	0.7	NS	2.0	NS	6.1	NS	2.7	NS	3.4	NS	3.4	NS
Participant with diagnosis of cancer	No	6.9	<0.05	1.0	NS	6.4	<0.01	3.8	NS	3.2	NS	4.3	NS	4.8	NS
	Yes	0.0	NS	0.0	NA	5.2	NS	10.5	NS	0.0	NA	5.3	NA	5.2	NS
Participant treated oncologically	No	6.0	NS	1.1	NS	4.8	<0.05	3.7	NS	3.2	NS	3.8	NS	4.3	NS
	Yes	9.5	NS	0.0	NA	19.0	NA	9.5	NS	0.0	NS	9.5	NA	9.5	NA
Self-declaration of sufficient cancer-related level of knowledge	No	5.6	NS	1.9	NS	5.5	<0.05	4.3	NS	5.0	NS	5.6	NS	6.2	<0.05
	Yes	8.7	NS	-2.2	NA	8.7	NS	4.3	NS	-4.4	NS	0.0	NS	0.0	NS
Self-declaration of willingness to improve the level of cancer-related knowledge	No	13.4	NS	0.0	NA	6.6	NS	13.4	NS	0.0	NS	-6.7	NS	-6.7	NS
	Yes	5.7	NS	1.1	NS	6.2	<0.01	3.6	NS	3.1	NS	5.2	NS	5.7	<0.05

D – relative difference between final and initial percentage of consents (in %); p – statistical significance in McNemar test; NS – not significant; NA - not available; BFNA - Breast Fine Needle Aspiration; USG – abdominal ultrasonography; Col–colonoscopy; Bron.–bronchoscopy; Pan. – panendoscopy.
